# Functional characterization of human Heschl’s gyrus in response to natural speech

**DOI:** 10.1016/j.neuroimage.2021.118003

**Published:** 2021-03-28

**Authors:** Bahar Khalighinejad, Prachi Patel, Jose L. Herrero, Stephan Bickel, Ashesh D. Mehta, Nima Mesgarani

**Affiliations:** a Mortimer B. Zuckerman Brain Behavior Institute, Columbia University, New York, NY, United States; b Department of Electrical Engineering, Columbia University, New York, NY, United States; c Hofstra Northwell School of Medicine, Manhasset, NY, United States; d The Feinstein Institutes for Medical Research, Manhasset, NY, United States

**Keywords:** Heschl’s gyrus, iEEG, Auditory field maps, Tonotopy, Cortical mapping, Human auditory cortex

## Abstract

Heschl’s gyrus (HG) is a brain area that includes the primary auditory cortex in humans. Due to the limitations in obtaining direct neural measurements from this region during naturalistic speech listening, the functional organization and the role of HG in speech perception remain uncertain. Here, we used intracranial EEG to directly record neural activity in HG in eight neurosurgical patients as they listened to continuous speech stories. We studied the spatial distribution of acoustic tuning and the organization of linguistic feature encoding. We found a main gradient of change from posteromedial to anterolateral parts of HG. We also observed a decrease in frequency and temporal modulation tuning and an increase in phonemic representation, speaker normalization, speech sensitivity, and response latency. We did not observe a difference between the two brain hemispheres. These findings reveal a functional role for HG in processing and transforming simple to complex acoustic features and inform neurophysiological models of speech processing in the human auditory cortex.

## Introduction

Heschl’s gyrus (HG), also known as the transverse temporal gyrus, is an important part of the human auditory cortex. HG has been suggested as the location of the core auditory area because the cellular structure (koniocortex) and myelination of HG in postmortem tissue indicate dense thalamic input from the medial geniculate body (Brodmann, 1909; Campbell, 1905; Hackett, 2015; Hackett et al., 2001). While these architectonic studies suggest that the primary auditory cortex (PAC) is located in HG, this area is functionally heterogeneous, contains multiple auditory fields (Clarke and Morosan, 2012), and has high morphological variability across individuals and brain hemispheres (Rademacher et al., 2001). Neurophysiological models of speech processing in the human auditory cortex have postulated a limited role for HG in processing low-level acoustic features (DeWitt and Rauschecker, 2012; de Heer et al., 2017; Hickok and Poeppel, 2007). However, due to the limitations in obtaining direct neural measurements from the human auditory cortex, the functional organization and the role of HG in speech perception remain uncertain.

Auditory field maps have been extensively studied in HG using both noninvasive (fMRI) and invasive (iEEG) neurophysiology techniques. These studies have examined the organization of temporal modulation, frequency tuning, response latency, and speech sensitivity in HG. Stimuli such as modulated tones were used to measure temporal modulation tuning in HG to reveal a medial to lateral gradient, both with fMRI (Herdener et al., 2013; Leaver and Rauschecker, 2016; Overath et al., 2012; Santoro et al., 2014; Schönwiesner and Zatorre, 2009) and iEEG (Brugge et al., 2009). Moreover, response latency has been shown to increase from the posteromedial to anterolateral part of HG when listening to click trains and syllables (Nourski et al., 2014). Studies of tonotopic maps, on the other hand, have not produced consistent results. There are multiple proposed orientations of the tonotopic map in HG, which include perpendicular to HG (Besle et al., 2019), parallel to HG (Wessinger et al., 1997), circular (Barton et al., 2012), and high- low-high frequency gradient along the posteromedial to anterolateral axes (Dick et al., 2012; Moerel et al., 2014; Saenz and Langers, 2014). Responses to pure tones recorded with iEEG have also shown a high- to low-frequency tuning gradient within posteromedial HG (Howard et al., 1996; Nourski, 2017). Possible causes for these discrepancies could be the limited temporal resolution of fMRI, different types of synthetic stimuli used, and the limited coverage of HG in iEEG studies (Nourski et al., 2014). It has also been shown that the lateral part of HG responds more to speech than other sounds (Billig et al., 2019; Brugge et al., 2009; Moerel et al., 2012; Norman-Haignere et al., 2015) and the posteromedial part of HG shows different encoding of temporal modulation of speech than anterolateral HG (Billig et al., 2019; Nourski et al., 2009).

While these studies each sought to isolate and study individual tuning dimensions in HG, collectively, they reveal that sound encoding in HG is multifeatured and varies across several overlapping dimensions that include frequency, temporal modulation, and response latency. In addition, these studies have shown that HG cannot be considered a generic low-level sound processor. Instead, HG contains specialized and distinct computations for processing speech sounds. As a result, synthetic and simple stimuli may only partially activate the neurons in HG (Bitterman et al., 2008; Hamilton and Huth, 2018; Theunissen et al., 2000) and fail to fully capture the integrated and interactive encoding of simultaneously varying acoustic dimensions that coexist in a naturalistic sound such as speech (Bitterman et al., 2008; Hamilton and Huth, 2018; Theunissen et al., 2000). As a result, much remains unclear regarding the multifeatured functional organization of HG, the relationship between different tuning maps, and the emergence of speech-specific properties in HG. More specifically, I) what is the role of HG in the transformation of acoustic to phonetic features? Is there a change in speech sensitivity, speaker normalization and invariant phonemic representation across HG? These questions are important, as inconsistent results have been reported regarding the transformation from acoustic to phonemic features, which is reported to exist both outside of HG (Turkeltaub and Coslett, 2010) and extend into the core (Woods et al., 2011). II) Are the characteristic maps in HG orthogonal to each other as observed in mammalian auditory cortex (Bizley et al., 2009; Mesgarani et al., 2008; Walker et al., 2011) or similar to those reported in human superior temporal gyrus (Hullett et al., 2016)? III) Is there a difference between tuning properties in the left and right HG, as suggested by recent studies showing lateralized asymmetry in temporal modulation processing (Albouy et al., 2020; Flinker et al., 2019)?

To answer these questions, we used iEEG to directly measure neural activity in a cohort of 8 neurosurgical patients with comprehensive coverage of left and right hemisphere HG. We measured the neural responses as the patients listened to natural speech stories. We studied the multidimensional tuning properties of HG in response to various acoustic and linguistic attributes and measured the organization of neural responses along several tuning dimensions to create high-resolution maps of response tuning to individual and joint acoustic and linguistic attributes. Specifically, the characteristic maps for best frequency, response latency, temporal modulation, speaker invariance and speech sensitivity were created.

## Methods

### Intracranial recordings

Eight adults (five females) with pharmacoresistant focal epilepsy were included in this study. All subjects underwent chronic intracranial encephalography (iEEG) monitoring at North Shore University Hospital to identify epileptogenic foci in the brain for later removal. All subjects were implanted with depth electrode arrays. All subjects were between 18 and 60 years old. All subjects were fluent speakers of American English and had self-reported normal hearing. In seven subjects, the left hemisphere, and in one subject, the right hemisphere were dominant for language (as determined with the Wada test). Electrodes showing any sign of abnormal epileptiform discharges, as identified in epileptologists’ clinical reports, were excluded from the analysis. iEEG time series were manually inspected for signal quality and were free of interictal spikes. All research protocols were approved and monitored by the institutional review board at the Feinstein Institute for Medical Research, and informed written consent to participate in research studies was obtained from each subject before implantation of electrodes. Bifurcations in Heschl’s gyrus (HG) were detected by neurologists and neurosurgeons. Subjects who had bifurcations are shown in Table 1. The electrode locations were selected by the clinical team for the clinical needs of the patient. The age, sex, language laterality determined using the Wada test, seizure focus, and number of contacts in each HG are shown in Table 1. All subjects learned the English language before the age of 5 and have been using it for everyday interactions since then, thus making them fluent speakers. 3 subjects were bilingual, 2 monolingual and 3 had English as primary language but elementary or intermediate proficiency in an additional language.

### Data preprocessing and hardware

Intracranial EEG (iEEG) signals were acquired continuously at 3 kHz per channel (16-bit precision, range ± 8 mV, DC) with a data acquisition module (Tucker-Davis Technologies (TDT), Alachua, FL, USA). Either subdural or skull electrodes were used as references, as dictated by recording quality at the bedside after online visualization of the spectrogram of the signal. Speech signals were recorded simultaneously with the iEEG for subsequent offline analysis. All further processing steps were performed offline. The iEEG data were resampled to 500 Hz. A 1st-order Butterworth high-pass filter with a cutoff frequency at 1 Hz was used to remove DC drift. Line noise at 60 Hz and its harmonics (up to 240 Hz) were removed using 2nd-order IIR notch filters with a bandwidth of 1 Hz. A period of silence lasting two minutes was recorded before the experiments, and all the data were normalized by subtracting the mean and dividing by the standard deviation of this prestimulus period.

The envelope of high-gamma activity, which correlates with neural firing in the proximity of electrodes (Buzsáki et al., 2012; Ray and Maunsell, 2011), was used as a measure of the neural response. To obtain the envelope of this broad-band signal, we first filtered the data into eight frequency bands between 70 and 150 Hz. Then, the envelope of each band was obtained by taking the absolute value of the Hilbert transform. We took the average of all eight frequency bands as the final envelope.

### Stimulus and auditory spectrogram

All stimuli were presented using a single Bose SoundLink Mini 2 speaker situated directly in front of the subject. To reduce the inevitable acoustic noise encountered in uncontrolled hospital environments, all electrical devices in the patients’ room were unplugged except the recording devices, and the door and windows were closed during the experiment to prevent interruption.

All subjects listened to speech material containing short stories. Subjects 1 and 2 listened to stories recorded by two voice actors (one male and one female voice actor) with a duration of 25 min and a sampling rate of 16 kHz. Subjects 3 to 8 listened to different stories recorded by four voice actors (stories B: two male and two female voice actors, two of the speakers were common between the two tasks played for subjects 1 and 2 and for subjects 3–8) with a duration of 20 min and a sampling rate of 11,025 Hz. The sampling frequency of our stimulus (11,025 Hz) limits the maximum best frequency that can be measured to 5.5 kHz. However, this limitation is not a major concern as speech has relatively little power above 5 kHz (Dunn and White, 1940). Male 1, male 2, female 1, and female 2 have average absolute pitches of 92 Hz, 104 Hz, 174 Hz, and 191 Hz, respectively. The stories were played at a comfortable volume customized for each patient. The results were consistent when the responses to the two-speaker stimuli and four-speaker stimuli were analyzed separately.

The time-frequency representation of speech sounds was estimated using a model of cochlear frequency analysis (Yang et al., 1992) consisting of a bank of constant 128 asymmetric filters equally spaced on a logarithmic axis. The filter bank output was subjected to nonlinear compression, followed by a first-order derivative along the spectral axis (modeling an inhibitory network) and finally an envelope estimation operation. This resulted in a two-dimensional representation simulating the pattern of activity on the auditory nerve (Chi et al., 2005). The output of the filter bank was then resampled to 16 bands.

### Neural spectrotemporal receptive fields

**Fig 1.**
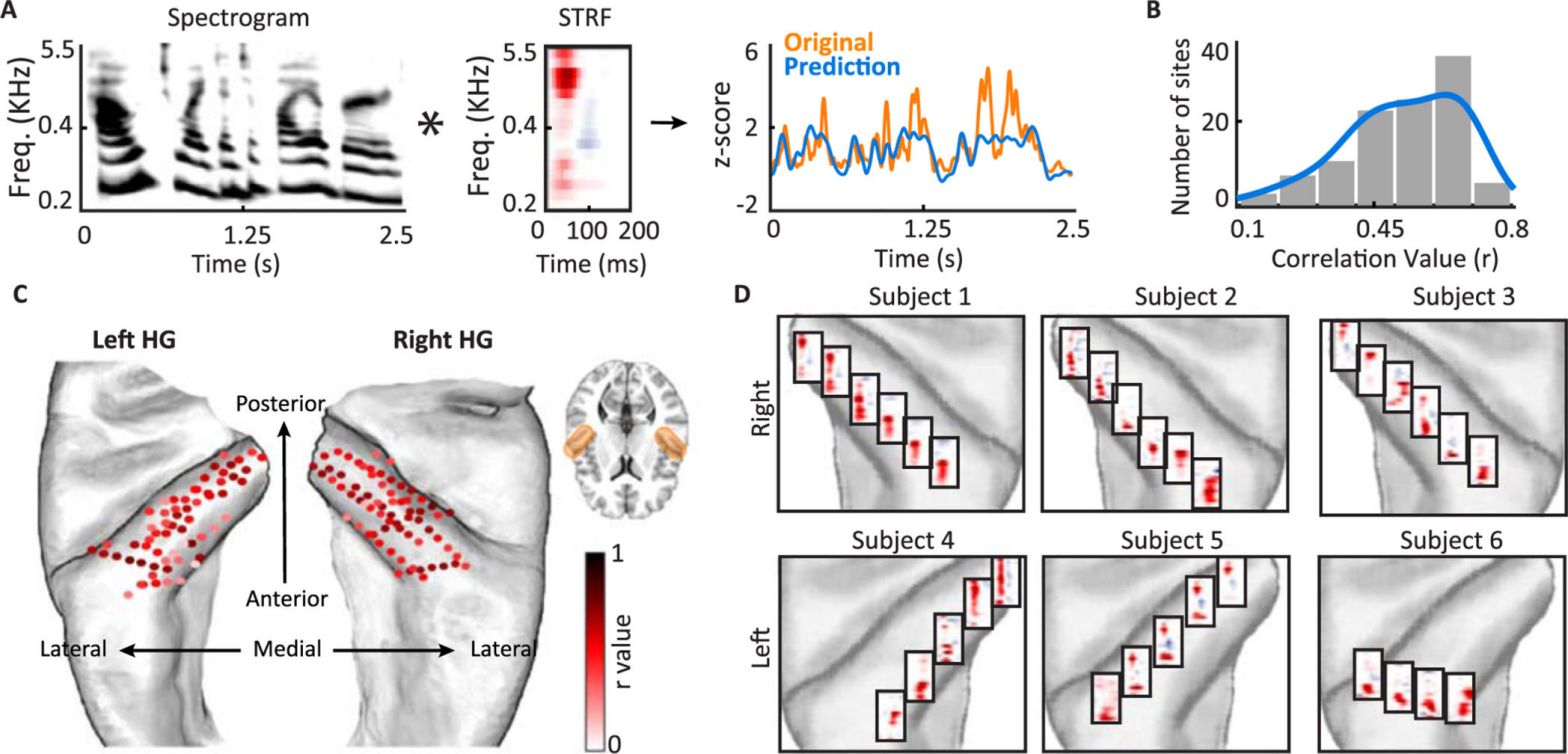
Spectrotemporal receptive fields (STRFs) calculated from 132 sites in Heschl’s gyrus (HG). A) Natural speech stories were played to the subjects, and the spectrotemporal receptive field (STRF) was calculated for each electrode. B) The histogram of correlation values between predicted and actual responses across electrodes. C) The locations of electrodes on Heschl’s gyrus and sulcus are shown on an average FreeSurfer brain. The colors indicate the correlation values between predicted (20-fold cross validation) and actual response. D) STRFs of 32 example electrodes are shown for six subjects on the left (bottom row) and right (top row) HG.

Using the speech stimulus and high-gamma activity recorded from the implanted electrodes, we measured the spectrotemporal receptive field (STRF) of each site (Theunissen et al., 2001a). STRF is defined as a filter that predicts the neural responses from the stimulus spectrogram (Fig. 1A). STRFs were computed by using the normalized reverse correlation algorithm using STRFLab (Theunissen et al., 2001b). Regularization and sparseness parameters were found via cross-validation. The best frequency and response latency parameters were estimated by finding the center of the excitatory region of the STRF along the frequency and time dimensions (Fig. S1). The best frequency and response latency measured using high-gamma activity were highly correlated with those measured from LFP (Fig. S2). The best temporal modulation parameter was estimated from the two-dimensional wavelet decomposition of the STRF. Wavelet decomposition extracts the power of the filtered STRFs at different temporal modulations (rates) (Chi et al., 2005; Mesgarani et al., 2006). The modulation model of STRFs has four dimensions: scale, rate, time, and frequency. To estimate the best temporal modulation, we first averaged the model over three dimensions of time, frequency, and scale to calculate a rate vector. Next, we found the weighted average of the rate vector, where weights are the rate values.

### Electrode inclusion criteria

The neural sites with significant STRF prediction accuracy (Pearson correlation) were included in all subsequent analyses (*t* -test, false discovery rate [FDR] corrected, Benjamini and Yekutieli, 2001, *p* < 0.01, *N* = 20). This selection criterion resulted in 132 electrodes in Heschl’s gyrus and sulcus across all subjects (68 electrodes in the right hemisphere, 64 electrodes in the left hemisphere). Using these 132 electrodes, the STRFs showed an average prediction correlation of 0.44 ± 0.10 SD (Fig. 1B). The location of electrodes on an average brain is shown in Fig. 1C.

### Speech sensitivity stimuli

To quantify the speech sensitivity of each neural site, six of the subjects (subjects 1, 3, 5, 6, 7, and 8) also performed a speech-nonspeech task (total of 85 electrodes). Subjects passively listened to 30 min of audio containing 69 commonly heard sounds (Fig. S3). The sounds consisted of coughing, crying, screaming, different types of music, animal vocalization, laughing, syllables, sneezing, breathing, singing, shooting, drum playing, subway noises, and speech by different speakers. In total, we had 53 unique nonspeech and 16 speech sounds, each presented once (Figs. S3 and S4). The stimulus variety was chosen to cover a broad range of spectrotemporal features using every day sound categories (Fig. S3), and corresponding sounds were downloaded from multiple corpora available online (e.g. BBC news speech corpus, RWC music database and freesound.org). All the 69 sounds used for this task are provided as supplementary material. The trials were on average 13.5 s long, and a silence duration of 1 s was added between consecutive trials. The neural data were preprocessed as explained in the “data preprocessing and hardware” section. To determine the speech sensitivity index, we first normalized the response of each site using the mean and variance of the neural data during the silent interval. We then averaged the normalized responses over the presentation of each sound. Finally, we performed an unpaired *t*-test between the averaged responses of all speech and all nonspeech sounds to obtain a *t*-value for each site denoting the specificity to speech over nonspeech sounds.

### Brain maps

Electrode positions were mapped to brain anatomy using registration of the postimplant computed tomography (CT) to the preimplant MRI via the postop MRI (Groppe et al., 2017). After coregistration, electrodes were identified on the postimplantation CT scan using BioImage Suite (Papademetris et al., 2006). Following coregistration, the electrodes were snapped to the closest point on the reconstructed brain surface of the preimplantation MRI. We used FreeSurfer automated cortical parcellation (Fischl et al., 2004) to identify the anatomical regions in which each electrode contact was located within approximately 3 mm resolution (the maximum parcellation error of a given electrode to a parcellated area was < 5 voxels/mm). We used Destrieux’s parcellation, which provides higher specificity (Fischl et al., 2004) in the ventral and lateral aspects of the medial temporal lobe (Destrieux et al., 2010) compared to Desikan–Killiany parcellation (Desikan et al., 2006). Automated parcellation results for each electrode were closely inspected by the neurosurgeon using the patient’s coregistered postimplant MRI. We mapped each electrode from an individual subject’s brain to a standard probabilistic atlas of the human brain from 152 human subjects, which captures intersubject variabilities such as bifurcations and duplications (Mazziotta et al., 2001). All contacts localized in HG by FreeSurfer (Destrieux et al., 2010; Fischl et al., 2004) and verified from individual patient CT by neurosurgeons were included in the analysis.

**Fig. 2.**
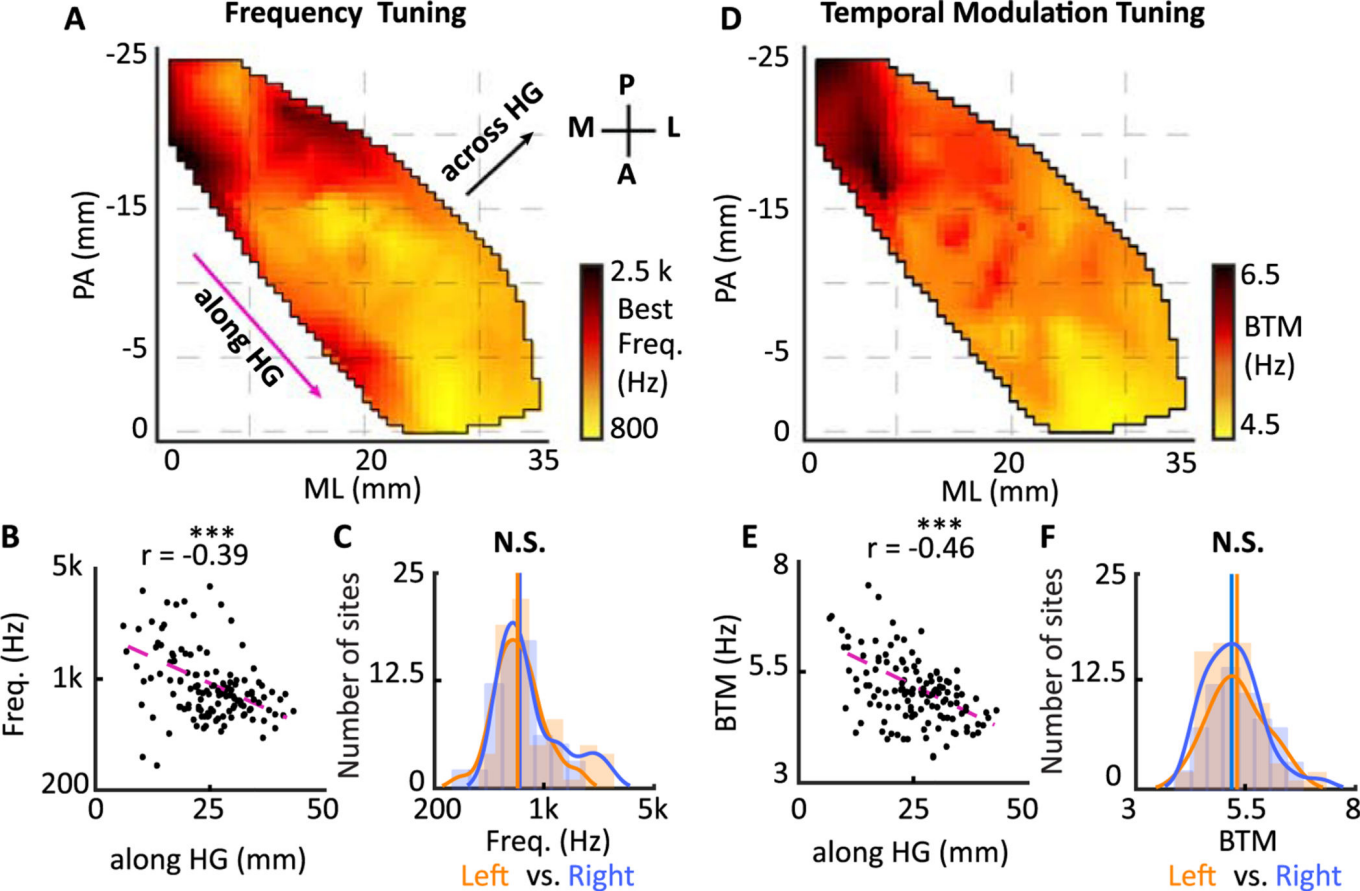
Spatial organization of frequency tuning and temporal modulation tuning. A) Spatial organization of frequency tuning is shown along two dimensions: medial to lateral (ML) and posterior to anterior (PA). B) Scatter plot of location along HG versus characteristic frequency of individual electrodes is shown (*Y*-axis is logarithmic) (Pearson correlation = −0.39, *t* -test, *p* < 0.001, *N* = 132). C) Histograms of characteristic frequencies estimated from neural sites in left and right HG (N.S. *P* > 0.1, *N*_*left*_ = 64*, N*_*right*_ = 68, Wilcoxon rank-sum test). D) Spatial organization of temporal modulation tuning. E) Scatter plot of location along HG versus temporal modulation tuning of sites (Pearson correlation = −0.46, *t*-test, *p* < 0.001, *N* = 132). F) Histograms of best temporal modulation (BTM) tuning estimated from sites in left and right HG (N.S. *P* > 0.1, *N*_*left*_ = 64*, N*_*right*_ = 68, Wilcoxon rank-sum test).

**Fig. 3.**
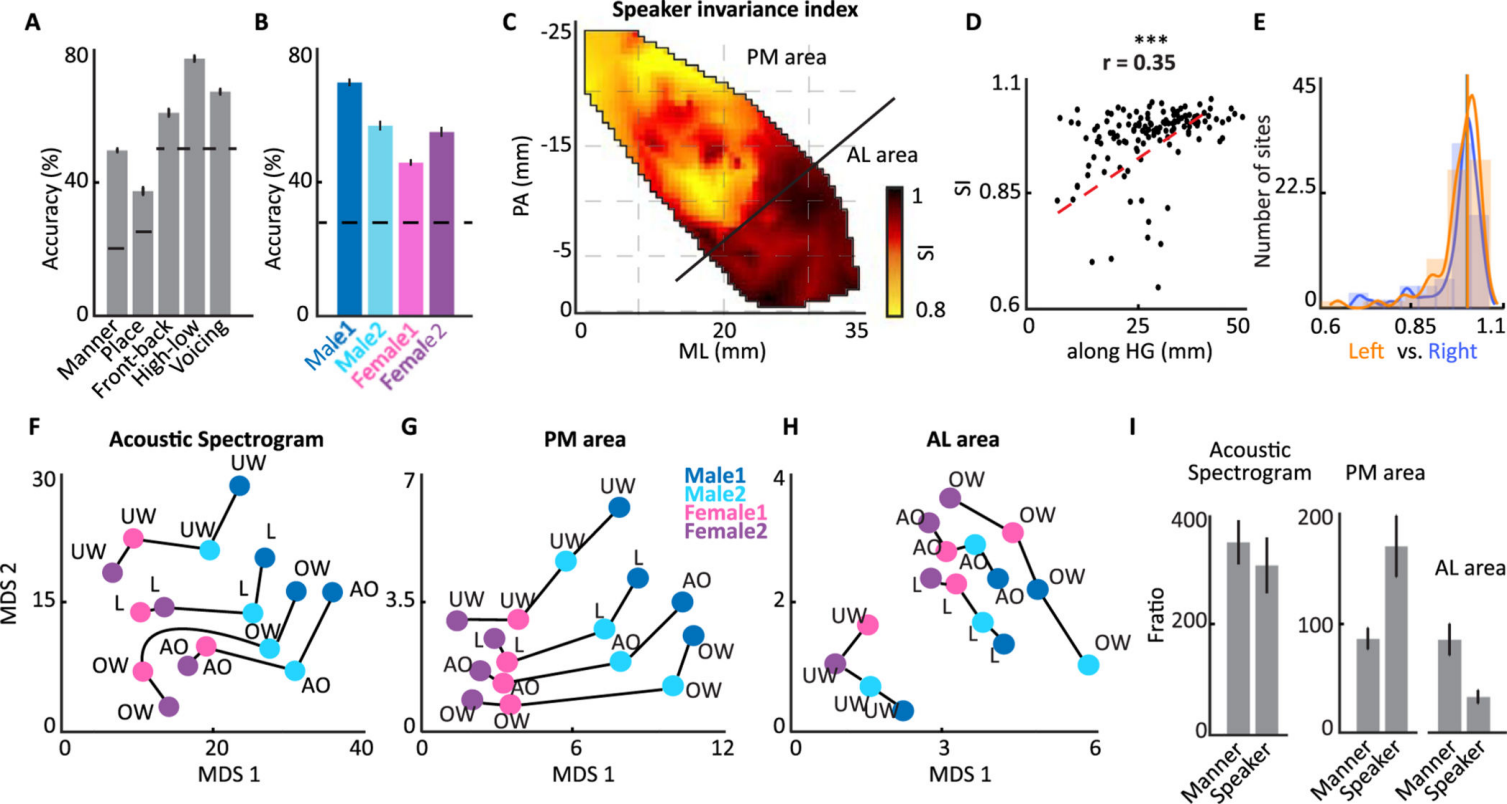
Spatial organization of the speaker invariance index in HG. A) Classification accuracy of different phonetic features using the population of neural sites in HG. Horizontal lines display chance performance. The error bars depict standard error. B) Classification accuracy of different speakers using populations of neural sites in HG. Horizontal lines display chance performance. C) Characteristic map of the speaker invariance index on two dimensions of ML and PA. The *boundary* is the anterior one-third vs posterior two-thirds. D) Speaker invariance index versus location along HG for individual electrodes (Pearson correlation = 0.35, *t*-test, *p* < 0.01, *N* = 132). E) Histograms of the speaker invariance index estimated from neural sites in left and right HG (N.S. *P* > 0.1, *N*_*left*_ = 64*, N*_*right*_ = 68, Wilcoxon rank-sum test). F–H) MDS diagram of four phonemes spoken by four speakers derived from the acoustic spectrograms, the population neural responses in the PM (posteromedial) part of HG, and in the AL (anterolateral) part of HG. I) *F*-ratio distance between four speakers vs *F*-ratio distance between five manners of articulation in acoustic spectrograms, PM area, and AL area. The error bars depict standard error.

**Fig 4.**
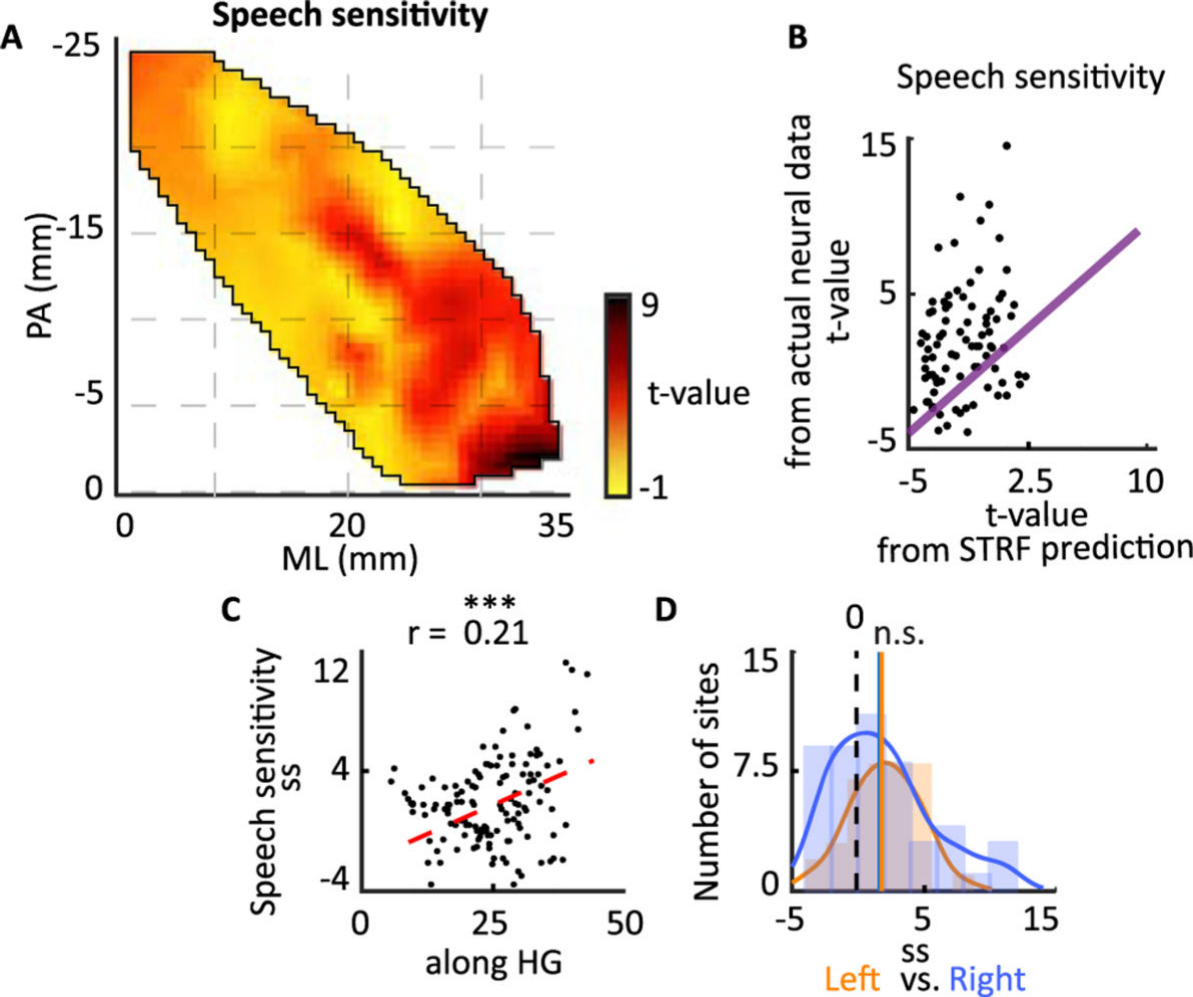
Spatial organization of speech sensitivity. A) Spatial organization of speech sensitivity. B) Comparison of speech sensitivity calculated from STRF predictions versus actual neural data. C) Scatter plot of location along HG versus speech sensitivity of individual sites (Pearson correlation = 0.21, *t*-test, *p* < 0.05, *N* = 132). D) Histograms of speech sensitivity estimated from neural sites in left and right HG (N.S. P > 0.1, *N*_*left*_ = 37*, N*_*right*_ = 48, Wilcoxon rank-sum test).

**Fig. 5.**
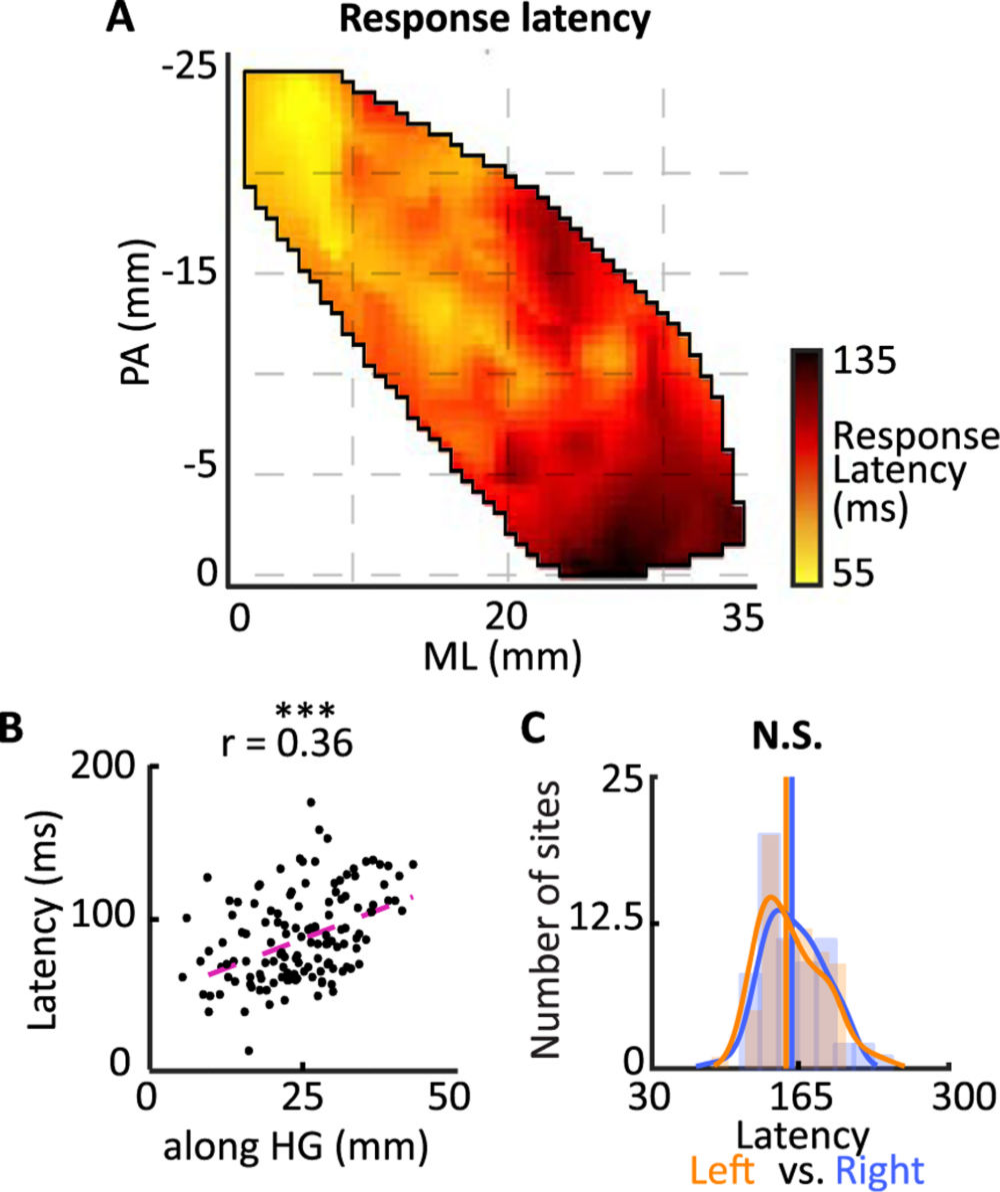
Spatial organization of response latency. *A) Spatial organization of response latency. B) Scatter plot of location along HG versus response latency of individual electrodes (*Pearson correlation = 0.36, *t*-test, *p* < 0.001, *N* = 132)*. C) Histograms of response latencies estimated from neural sites in left and right HG (N.S. P* > *0.1, N*_*left*_ = 64*, N*_*right*_ = 68*, Wilcoxon rank-sum test).*

To calculate the topographic feature maps for each hemisphere (Fig. S5), we used spatial smoothing for each tuning feature. Smoothing was performed by assigning the average of the four closest neighboring electrodes to each site using k-nearest neighbor search (KNN) (Euclidean distance). After smoothing, a piecewise linear interpolation surface was fitted to the values of sites using the MATLAB fit function. To find the combined map for both hemispheres (Figs. 2A, D, 3C, 4A and 5A), the distance of each site to the midsagittal plane was calculated using BioImage Suite (Papademetris et al., 2006). The absolute value of the distance to the midsagittal plane was used as the ML (medial to lateral) distance. For the purpose of readability, we set the minimum ML distance to zero. Spatial smoothing was only performed for visualization of the maps, but all significance tests and scatter plots were calculated using the actual raw values.

For the speech sensitivity map shown in Fig. 4A, in addition to the above steps, we also used the method of KNN imputation to fill the values of sites using the nearest neighbor (i.e. *k* = 1) for the two subjects for which the speech sensitivity task was not played. This method was only used for the purpose of visualization in Fig. 4A, and all the significance tests, correlations, and scatter plots were calculated using the actual values without KNN imputation and smoothing. The along HG distance was calculated by projecting electrode locations onto their first principal component (shown by the purple arrow in Fig. 2A).

### Phonemes analysis

We segmented the speech material and neural responses into time-aligned sequences of phonemes using the Penn Phonetics Lab Forced Aligner Toolkit (Yuan and Liberman, 2008). The spectrograms were aligned to the onset of phonemes with a time window of 200 ms previously shown to encode phonetic features in HG (Khalighinejad et al., 2019). To minimize the preprocessing effects, we did not normalize the natural variation in phoneme length.

### MDS diagram of phonemes

To calculate the MDS (multidimensional scaling algorithm) diagram of phonemes for each speaker using acoustic spectrograms (shown in Fig. 3F), we first found the average acoustic spectrogram of all instances of each phoneme spoken by each speaker. Next, the average phoneme spectrogram was windowed between 10 ms and 70 ms after the onset of the phoneme to restrict the time window to a smaller segment that incorporated acoustic differences in the phonetic categories. The duration of the window was chosen according to the maximum peak of the *F-*statistic between the categories of phonemes using all speakers (Khalighinejad et al., 2017a; Mesgarani et al., 2014). The *F*-statistic is a metric used to measure phonetic discriminability as a ratio of between-class variance to within-class variance (Patel et al., 1976). Next, we calculated the pairwise Euclidean distance between phonemes, which resulted in a two-dimensional symmetric matrix reflecting a pattern of pairwise phoneme dissimilarities. To visualize this dissimilarity matrix, we used a two-dimensional MDS using Kruskal’s normalized criterion to minimize stress for the two MDS dimensions (Cox and Cox, 2008). The MDS diagram of phonemes based on neural networks (Fig. 3G, H) was calculated using the same method with two differences. First, each instance of a phoneme was based on the segmented neural high gamma response to the phonemes. Second, because of the time delay between the stimuli and the response, the window was set to 90 ms to 150 ms after the onset of phoneme. This window was chosen to maximize the *F*-statistic of the neural responses to phoneme categories (Khalighinejad et al., 2017a; Mesgarani et al., 2014).

### Classification of phonemes and speakers

To examine the encoding of both speakers and articulation features at the population level, we trained a regularized least square (RLS) classifier (Rifkin et al., 2003) to predict the speaker or articulation feature of individual instances of the time-locked evoked responses to phonemes (Khalighinejad et al., 2017a) (10% of data used for cross-validation). The input to the classifier was the concatenation of all of the neural sites in the HG area with a window of 70 ms to 180 ms after the onset of phoneme. One classifier was trained for subjects 1 and 2, and a separate classifier was trained for subjects 3 to 8 due to the different number of speakers that these subject groups heard. The results of both classifiers were consistent.

### Speaker invariance index

To study the categorical encoding of phonemes in HG, we examined the similarity of the neural response to various phonemes when uttered by different speakers. Specifically, we measured the degree of speaker normalization for each neural site using a phonetic feature classifier (RLS classifier) that was trained on three of the speakers and was tested on the unseen speaker. Because the baseline decoding accuracy depends on the signal-to-noise ratio across neural sites, we divided the phonetic feature classification accuracy for the unseen speaker by the classification accuracy when the classifiers were trained within each speaker. Within speaker phoneme classification was performed by training and testing the phoneme classifier using utterances from the same speaker (10% cross-validation). This normalized phonetic feature classification accuracy on the held-out speaker was defined as the degree of phonemic categorization, and we call it the speaker invariance index.

### Joint spatial organization of characteristic feature tuning

To establish a relationship between the five characteristic maps reported and to find the correlational structure of tuning to various characteristic attributes, we used the method of principal component analysis (PCA). PCA is a dimensionality reduction technique that combines the most correlated dimensions in the data. In our analysis, each characteristic map was considered a feature, and PCA was performed on *x* = (*x*_1_*, x*_2_, …*, x*_*M*_)′, where M (columns) is the number of characteristic maps, *x*_*i*_ are vectors of N points, and N (rows) is the number of neural sites. Therefore, the PCA computes the weighted sum of feature maps where weights indicate the correlation of feature maps across all neural sites. To find the dominant direction of change for each PC projection on Heschl’s gyrus, we used canonical correlation analysis.

Therefore, the plotted best direction represents vector *â*, where <di>, *X* is the concatenation of the ML (medial to lateral) and PA (posterior to anterior) distances, and *Y* is the projected tuning value on the PCs. As a control, assigning random values to *Y* resulted in vectors (∨) with random direction without preference for any specific site, confirming that the location of neural sites did not have any effect on the calculated best direction.

## Results

We measured five tuning attributes for each neural site: acoustic frequency, temporal modulation, speaker invariance index, speech-nonspeech response difference, and response latency. In all of the analyses, we used the envelope of the high gamma frequency band (70–150 Hz) as the measure of neural response (Buzsáki et al., 2012; Ray and Maunsell, 2011). We first present the result of the tuning analysis for each of the five tuning parameters separately and then show the properties of the joint tuning to all characteristic features. The spatial organization of neural responses to each characteristic feature for all subsequent analyses is shown along the two directions of PA and ML of HG using the location of electrodes on the average FreeSurfer brain (FreeSurfer template brain, ICBM152) (Fischl et al., 2004). Across all subjects, there were 132 electrodes in Heschl’s gyrus and sulcus. Analyzing all subjects together provided adequate coverage of different sections of HG along the axes of PA and ML (Fig. 1C). We did not observe a clear spatial organization for STRF prediction accuracy (Fig. 1C). Example STRFs are shown in six of the subjects in Fig. 1D, illustrating the diversity of tuning to spectral and temporal characteristic features. For the subsequent analysis in this paper, we combined the left and right HG to generate the characteristic maps.

### Spatial organization of best frequency

The best frequency (BF) parameter for each cortical site is defined as the location of the excitatory peak of the STRF along the frequency dimension (Theunissen et al., 2001a). The spatial map of the BF shows multiple areas with tuning to low and high frequencies, including a high- low-high gradient of frequency selective areas creating a “V”-shaped pattern across HG and a high- to low-frequency gradient on the anterolateral part of HG (Fig. 2A). Despite the mixed frequency tuning patterns in HG, we found an overall gradient of high- to low-frequency tuning that extends from the posteromedial to anterolateral region. This gradient is shown in Fig. 2B, where the distance from the posteromedial part of HG (along HG distance) is plotted against the frequency tuning for individual electrodes. The along HG distance is calculated by projecting electrodes’ location onto the HG axis (shown with a purple arrow in Fig. 2A) and measuring the distance from the most medial location on HG. The high- to low-frequency gradient along HG was similar in both left and right HG (Figs. S5 and S6), and there was no significant difference between the distribution of the best frequency between the left and right hemispheres (Wilcoxon rank-sum test, *P* > 0.1, *N*_*left*_ = 64*, N*_*right*_ = 68, Fig. 2C).

### Spatial organization of best temporal modulation

In addition to the best frequency, the best temporal modulation can also be calculated from the STRF. Temporal modulation distinguishes between slowly and rapidly changing characteristic features. The human perception of sound is highly sensitive to a wide range of temporal modulations (Theunissen and Miller, 1995; Viemeister, 1979), and this acoustic attribute has been shown to be an organizing factor in the human auditory cortex (Hullett et al., 2016). We defined the best temporal modulation (BTM) of a site as the peak of its STRF wavelet decomposition along the time axis (Woolley et al., 2005), where the transformed time axis is referred to as the rate (in Hz). The differences between STRFs with different rates are shown with seven examples (slow to fast) in Fig. S1. The spatial organization of temporal modulation tuning in HG is shown in Fig. 2D. The significant correlation of temporal modulation tuning with HG distance in Fig. 2E illustrates an increase from the posteromedial to anterolateral region. There was no significant difference between the distribution of temporal modulations in the left versus right Heschl’s gyrus (Wilcoxon rank-sum test, *P* > 0.1, *N*_*left*_ = 64*, N*_*right*_ = 68, Fig. 2F). It is also worth mentioning that the values and maps shown in Fig. 2 were consistent when the STRFs were estimated from two nonoverlapping subsets of stimulus-response pairs (test–retest), indicating the reliability of the STRF measurements across stimuli and their robustness to varying degrees of neural noise (Figs. S7 and S8).

### Spatial organization of speaker invariance index

In the previous sections, we studied the organization of the best frequency and temporal modulation. However, understanding human speech is not only dependent on acoustic attributes but also is dependent on the successful decoding of linguistic units. Phonemes are the smallest contrastive units in a language to which the auditory cortex responses show some large-scale spatial organization (Fishman et al., 2016; Khalighinejad et al., 2017a; Di Liberto et al., 2015; Mesgarani et al., 2014; Steinschneider et al., 2004). One of the major sources of acoustic variability in phones (each instance of phonemes) within the same phoneme category is the difference between different speakers’ voices. Normalizing speaker variability is crucial for robust decoding of the phoneme category and therefore the spoken message. At the same time, representing speaker variability is necessary for the successful identification of speakers. Previous studies have shown that a categorical representation of phonemes appears in higher-level cortical areas where the encoding of phonemes becomes less sensitive to perceptually irrelevant acoustic variations (allophones) (Chang et al., 2010; Formisano et al., 2008). This emergence of phoneme categories, however, has not been studied in HG.

To examine the extent to which different phonemes and speaker identities are represented in HG, we used the phonetic transcription of speech utterances to obtain time-aligned neural responses to all instances of each phoneme. We first quantified the separability of phonemes and speakers using a linear classifier trained to decode phonetic features and speaker identities (10% cross-validation was used). We restricted the analysis to five representative phonetic attributes that fully measure encoding of the amount of phonetic information. These were the manner and place of articulation features, high-low and front-back vowel distinctions, and a voiced-unvoiced attribute (Mesgarani et al., 2014). We found that we could successfully decode all five phonetic features significantly higher than chance from the population of HG responses (Fig. 3A). To estimate the encoding of speaker differences in HG, we classified the identity of the four speakers based on neural responses to individual phonemes. We found that the speaker differences were also decodable significantly above chance in the population responses (Fig. 3B). The confusion matrices for classification of both the phonetic features and the speaker identity are shown in Fig. S9.

To find the degree of phoneme encoding at each neural site, we defined a speaker invariance index (SI) that measures the invariance of phoneme encoding to different speakers (details in methods). We found that categorical phoneme encoding increased towards the anterolateral part of HG. The spatial organization of speaker-invariant phoneme encoding in HG is shown in Fig 3C. This figure shows two distinct encoding schemes in the anterolateral and posteromedial parts of HG. The majority of sites in anterolateral HG (AL area in Fig. 3C) show a higher categorical and less speaker-dependent encoding of phonemes compared to the posteromedial part of HG (PM area in Fig. 3C). This analysis shows an increase in categorical representation of phonemes towards the anterolateral part of HG. Similar to the previous maps, phoneme encoding was also significantly correlated with the along HG distance of electrodes (Fig. 3D, *r* = 0.35). There was no difference between the degree of phonemic encoding and speaker normalization between left and right HG (Wilcoxon rank-sum test, *P* > 0.1, *N*_*left*_ = 64*, N*_*right*_ = 68, Fig. 3E).

To further illustrate the population encoding of phonemes and speakers in posteromedial and anterolateral HG (shown in Fig. 3C), we examined the relative distance between four representative phonemes of /UW/, /L/, /AO/, and /OW/ spoken by all four speakers. We used the first two multidimensional scaling (MDS) dimensions of phoneme responses to express their relative distances in the acoustic space and in the neural space (Fig. 3F–H). The population response in posteromedial HG shows a clear separation between the responses to different phonemes and speakers (Fig. 3G), similar to the representation of phonemes in acoustic space (Fig. 3F). The four speakers are shown with different colors in the MDS diagram. In contrast to posteromedial HG, the population responses in anterolateral HG (Fig. 3H) still group the phonemes of the same category together, but the separation between the phonemes of the four speakers is no longer preserved. This effect can be quantified for all phonemes and neural sites using the discriminability of the phoneme’s manners of articulation and speaker identities for acoustic spectrograms and the population of sites in the PM area and AL area (the MDS diagram for all phonemes is shown in Fig. S10). We observed that while the discriminability (defined as the *F*-ratio, Patel et al., 1976) of speaker identities and manner of articulation is similar in acoustic space, the discriminability of speaker identities is significantly higher than the discriminability of manner of articulation in posteromedial HG, whereas the exact opposite is true for anterolateral HG (Fig. 3I). Together, these results show that the anterolateral part of HG encodes a more categorical representation of phonemes by normalizing the difference between speaker voices. In comparison, posteromedial HG strongly encodes speaker-specific differences.

### Spatial organization of speech sensitivity

We observed that phonemic encoding increases towards the anterolateral region of HG. Since phonemes are specific to human speech, we tested whether anterolateral sites in HG also respond preferentially to speech over non-speech sounds (Chan et al., 2013; Moerel et al., 2012; Norman-Haignere et al., 2015). We defined the speech sensitivity of a neural site as the t-value of a *t*-test between the average response of the site to speech versus nonspeech sounds (Chan et al., 2013), which in our study consisted of 69 sounds (53 non-speech, 16 speech) from 14 categories (Fig. S3). The speech sensitivity values are shown on HG in Fig. 4A which shows the highest values on the anterolateral side of HG. We found that 39% of electrodes in HG were significantly more responsive to speech than other sounds (*t*-test, FDR corrected, *q* < 0.01). To confirm that speech sensitivity is not a consequence of simple acoustic tuning and requires nonlinear transformation of the sound, we compared the actual and predicted speech sensitivity using electrodes’ STRFs. The speech sensitivity calculated from actual neural data was significantly higher than speech sensitivity calculated from STRF predictions (Fig. 4B). This shows the failure of the linear STRFs to account for the speech sensitivity of sites, and confirms that this response characteristic requires nonlinear signal processing which simpler acoustic attributes such as frequency and temporal modulation tuning cannot account for.

We examined the spatial organization of speech sensitivity using its correlation with along HG distance of electrodes. The significant correlation shown in Fig. 4C (*r* = 0.21) confirms a strong gradient of speech sensitivity from the posteromedial to anterolateral part of HG. Speech sensitivity in both the left and right Heschl’s gyrus was significantly higher than zero, and we did not observe any difference between speech sensitivity values in the left vs. right Heschl’s gyrus (Wilcoxon rank-sum test, *P* > 0.1, *N*_*left*_ = 37*, N*_*right*_ = 48, Fig. 4D).

### Spatial organization of response latency

The latency of the response along the auditory pathway approximately reflects the number of synapses away from the auditory periphery and hence has been used to speculate the direction of information processing in the auditory cortex (Da Costa et al., 2011; McMurray and Jongman, 2011; Nourski et al., 2014). We defined the response latency of neural sites as the excitatory peak of the STRF along the time dimension. The observed response latencies varied from 30 to 200 ms in different parts of HG, where it was lowest in the posteromedial part and gradually increased towards the anterolateral part (Fig. 5A). This gradient is shown in Fig. 5B, where latency is plotted against the HG location (left vs. right is shown in Figs. S5 and S6). There was no significant difference between the distribution of latency in the two hemispheres (Fig. 5C).

### Multivariate organization of characteristic feature tuning

**Fig. 6.**
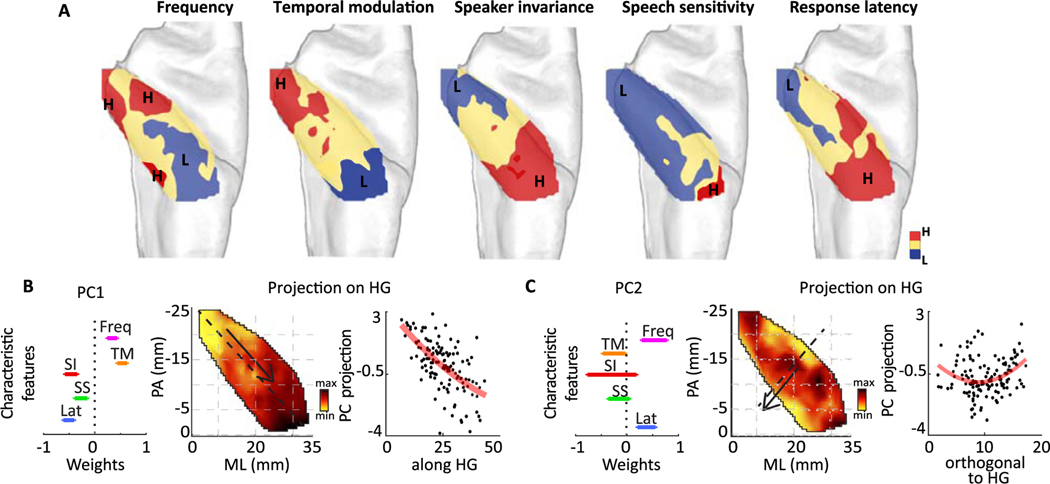
Joint spatial organization of characteristic feature tuning. A) Spatial organization of frequency tuning (Freq), response latency (Lat), temporal modulation (TM), speech sensitivity (SS) and phonemic encoding (PE). Plots are discretized to one-third of the maximum value to maximum and one-third of the minimum value to the minimum. B) The first principal component of joint tuning maps. The weights of the first PC are shown on the left (error bars indicate standard error calculated by bootstrapping the neural sites), and projection of tuning parameters onto the first PC is shown for all sites (middle). The first PC projection versus location along the HG axis is shown on the right. Red curve is the binomial fit. C) The second principal component of characteristic maps: the weights of the second PC are shown on the left (error bars indicate standard error calculated by bootstrapping the neural sites), projection of tuning parameters onto the second PC is shown for all sites in HG (middle), and second PC projection versus location on the orthogonal HG axis is shown on the right. Red curve is the binomial fit.

Neurons in the auditory cortex have multidimensional and joint tuning to different characteristic attributes (King and Nelken, 2009; Walker et al., 2011). Our analysis thus far focused on the anatomical organization of tuning to individual characteristic attributes, as summarized in Fig. 6A. This figure shows a correlated multidimensional organization of tuning maps to individual characteristic features. Therefore, a joint analysis of the individual tuning maps can offer further and complementary evidence for the organization of auditory fields and the main gradients of tuning change in HG (Fig. S11).

We used an unsupervised approach to examine the organization of joint tuning and to determine the dominant anatomical directions of tuning changes in HG. We performed a principal component analysis (PCA) on tuning to all five acoustic attributes across the HG sites. The PCA therefore summarizes the correlation patterns among the tuning to individual acoustic attributes. We found that the first and second principal components of tuning values can account for 63% of the variance (40% and 23%, respectively; third and fourth PCs are included in Fig. S12). The weights of the first two PCs are shown in Fig. 6B,C. The first PC shows that across all HG sites, a positive correlation exists between tuning to frequency and temporal modulation, which is negatively correlated with response latency, speech sensitivity, and phoneme encoding (Fig. 6B, left). The projected tuning values on the first PC are shown in Fig. 6B for all HG sites, where the dominant direction of change is calculated using canonical correlation analysis. This analysis finds the linear combination of ML and PA distances that has the maximum correlation with projected tuning values on the PCs. This unsupervised method shows that the direction that best describes the joint functional maps runs along the axis of HG. Because the first PC assigns significant nonzero weights to all acoustic attributes, this direction can be interpreted as the main axis along which frequency and temporal modulation tuning increase, while latency, speech sensitivity, and speaker invariance decrease. The second PC shows the second main correlation pattern among the tuning maps and reveals a positive correlation between tuning to frequency and response latency. The projected attributes on the second PC are shown in Fig. 6C, where the direction of maximum change is orthogonal to the HG long axis, resulting in a secondary dominant axis of the tuning gradient in HG. We further controlled the effect of intersubject variability by first creating a standard deviation map across subjects for each characteristic map (Fig. S13) to show that there is an absence of a unified direction of change. Second, a linear mixed effects model analysis showed that the characteristic maps for best frequency, response latency and temporal modulation hold when controlling for individual subject identity (Fig. S14). The maps for speaker invariance and speech selectivity show greater dependence on particular subjects’ data. Moreover, the maps are not influenced by the responsiveness of the electrodes’ high gamma activity to the stimulus (Fig. S15).

## Discussion

We examined the spatial organization of multiple tuning attributes in human HG in response to continuous speech. We found specific spatial maps for frequency, response latency, temporal modulation, speech sensitivity, and phonemic encoding in HG. Our results suggest that the best frequency and temporal modulation tuning decrease in the posteromedial to anterolateral direction in HG. In contrast, the response latency, speech sensitivity, and phoneme encoding increased along this HG direction. We also analyzed the properties of the joint tuning to all acoustic attributes and showed two prominent directions that explain the majority of correlated tuning changes, one along the PM-AL axis of HG and the other orthogonal to the axis. Compared to previous studies that either used unnaturalistic stimuli such as tones (Howard III et al., 1996; Moerel et al., 2014), ripples (Leaver and Rauschecker, 2016), or consonant-vowel syllable stimuli (Steinschneider et al., 2011) or had limitations in the resolution of neural measurement methods (Moerel et al., 2012, 2014; Santoro et al., 2014, 2017), using direct intracranial recordings, our naturalistic speech stimuli revealed multidimensional feature tuning in HG that organizes the responses in this auditory region. Specifically, we could add insights into the characteristic maps for speaker invariance and categorical representation of phonemes in HG, the role of HG in transformation from simple to complex acoustic features of speech, and the relationship between the characteristic maps.

### Organization of characteristic frequency

Tonotopy, the spatial arrangement of frequency selectivity, is one of the fundamental organizing principles in the mammalian auditory cortex. Previous research that attempted to find the orientation of tonotopic maps in human HG is, however, inconclusive. Several fMRI studies that used tones and artificial stimuli showed multiple frequency-selective areas in HG. The cumulative evidence suggests that HG is located within a high-low-high gradient of frequency selective regions that create a “V”-shaped pattern. Beyond this main high-low-high frequency gradient, the orientation of this tonotopy and the number of frequency selective areas have been the subject of scientific debate (Barton et al., 2012; Brewer and Barton, 2016; Da Costa et al., 2011; Dick et al., 2012; Formisano et al., 2003; Humphries et al., 2010; Moerel et al., 2012, 2014; Phillips et al., 2000; Talavage et al., 2004; Thomas et al., 2015; Upadhyay et al., 2006). On the one hand, studies have reported a collinear orientation of a high-low-high frequency gradient along HG (Formisano et al., 2003; Woods et al., 2009), and on the other hand, studies have proposed that tonotopic progression runs perpendicularly across HG rather than parallel along HG (Da Costa et al., 2011; Humphries et al., 2010). Moreover, apart from the main high-low-high frequency gradient, an additional low-frequency region is often reported at the antero-lateral border of the main gradient on the anterior superior temporal sulcus or planum porale (Humphries et al., 2010; Moerel et al., 2012; Woods et al., 2009). Among the proposed maps of frequency tuning, our main result obtained from direct recordings mostly agrees with Moerel et al. (2014), which used tone pips and 7T fMRI measurements to report multiple subregions of low- and high-frequency selective areas in HG compared to the previously mentioned studies (Dick et al., 2012; Humphries et al., 2010). It is worth mentioning that we do see a trend of V-shaped low-high-low frequency selective areas, but recordings from a higher number of subjects might result in increased smoothing of frequency tuning maps and can highlight these effects, similar to a previous report (Dick et al., 2012).

### Organization of temporal modulation tuning

Several previous studies have shown an encoding of temporal modulations in the human auditory system (Herdener et al., 2013; Leaver and Rauschecker, 2016; Overath et al., 2012; Schönwiesner and Zatorre, 2009; Wang et al., 2011). Studies that used ripple stimuli showed that the preferred temporal rate was highest in medial HG (Herdener et al., 2013; Leaver and Rauschecker, 2016; Overath et al., 2012; Schönwiesner and Zatorre, 2009). These studies, however, did not provide a precise spatial organization of temporal modulation in HG. An organized representation of temporal modulation tuning has previously been reported in the superior temporal gyrus (Hullett et al., 2016). Here, we showed that HG also has a topographic representation of temporal modulation rates that decreases from posteromedial to anterolateral HG.

### Organization of speaker invariance index

The frequency and temporal modulation tuning measures were based on a linear model of stimuli-response relationship (the STRF model), which has been commonly used to characterize the tuning of auditory cortical neurons. The higher auditory cortical regions, however, become progressively more nonlinear (King and Nelken, 2009). The inadequacy of linear models in such cases necessitates complementary and model-independent methods to characterize response properties. To achieve this task, we extended our linear tuning framework by examining preferential tuning to speech and phoneme and speaker encoding. By measuring speaker invariance across phonetic features, we showed that speaker-invariant encoding of phonemes increases from posteromedial to anterolateral HG. This invariant encoding of phonemes suggests a processing step in creating categorical representations of phonemes in which the acoustic variability of phones imposed by different speakers is reduced. While previous studies have shown the emergence of categorical phoneme representation in the cortical surface of the superior temporal gyrus (STG) (Chang et al., 2010; Formisano et al., 2008; Mesgarani et al., 2014; Steinschneider et al., 2011), our results from depth electrodes suggest that phonemic representations also appears on the superior temporal plane. This is congruent with the studies that show that the anterolateral Heschl’s gyrus is as late in the hierarchy as the posterior parts of superior temporal gyrus (Nourski et al., 2014). A categorical representation of phonemes involves more than just speaker normalization. Other sources of variability, such as contextual and prosodic variations in phones, should also be normalized to form phonemic categories. This is particularly true for more confusable allophonic variation of phonemes that may not be fully resolved in an early processing stage such as HG. Comparison of phoneme normalization in HG, planum temporale (PT), and STG may shed light on the progressive appearance of these linguistic units.

### Organization of speech sensitivity

Specialization of the human auditory cortex for speech processing has long been established (Belin et al., 2000). Previous fMRI studies have shown that the lateral part of HG responds more to speech than to other sound categories and speech-like artificial stimuli (Moerel et al., 2012; Norman-Haignere et al., 2015). Our results showed that 40% of sites in HG responded preferentially to speech over nonspeech sound categories, and this speech sensitivity was highest in the anterolateral part of HG. Our observation supports the possibility that anterolateral HG might be a higher auditory field than posterior STG (Nourski et al., 2014). These findings are intriguing, particularly because the cytoarchitecture has shown the anatomical proximity and cytoarchitectonic similarity of lateral regions of Heschl’s gyrus to the medial regions (Hackett, 2007). Further research that allows for the joint analysis of anatomical and functional properties of human HG can result in a better definition of the core auditory cortex that is based on both functional and anatomical properties of the regions.

### Left and right hemisphere differences

Functional asymmetries in the human auditory cortex have long been debated in the field of neuroscience (Hickok and Poeppel, 2007). In initial reports of Broca and Wernicke areas, it was shown that damage to cortical regions in the left hemisphere impaired speech comprehension, but this was not the case when the damage was on the right side (Wernicke, 1874). It has also been shown that a lesion of the right HG disturbs sound localization performance on both sides of space, while this is not the case for the left HG (Zatorre and Penhune, 2001). Moreover, the neuroanatomy of the superior temporal plane shows asymmetry, where HG and PT are larger on the left side (Dorsaint-Pierre et al., 2006). In contrast to the historically established view of left lateralized speech comprehension, recent studies have argued for bilateral involvement of the STG in speech perception and production (Bozic et al., 2010; Cogan et al., 2014). While speech can be processed bilaterally, it does not rule out the possibility of functional and computational specialization in the left and right hemispheres. For example, recent studies showed differential activation of the left and right hemispheres, where temporal and spectral modulation processing was lateralized in the left and right hemispheres accordingly (Flinker et al., 2019). It has also been shown that asymmetric processing of temporal and spectral modulation will result in an asymmetric emergence of speech and music representation in the auditory cortex (Albouy et al., 2020). It is worth noting that these studies selectively filtered out spectral and temporal modulation of speech and music, resulting in synthetic and unnatural stimuli that may activate the auditory cortex differently (Overath et al., 2015). In contrast, we did not find any difference between the functional processing of left versus right HG in the processing of acoustic attributes. This lack of difference may suggest bilateral speech processing in HG (Hickok and Poeppel, 2007). Further research is needed to clarify whether the lateralization reported in previous studies (Albouy et al., 2020; Flinker et al., 2019) also occurs during naturalistic speech perception.

### Organization of response latency

The latency of the response at a neural site approximates the number of synapses that the neural response to sound has to travel before reaching that site. As such, we would expect a primary region such as the core auditory area to have shorter latencies in comparison with non-primary regions such as belt and parabelt areas. In nonhuman primates, it has been confirmed that caudal belt and parabelt areas have shorter response latencies than rostral areas (Camalier et al., 2012; Kajikawa et al., 2005). One advantage of using direct neural measurements in our study compared to fMRI is the ability to measure the response latency with a high degree of precision. We observed a wide range of response latencies in HG from 30 ms to 200 ms. Similar to the frequency map, the main orientation of latency increase runs along the PM to AL axis of HG. This result supports the notion that the primary auditory cortex is located in the posteromedial part of HG. Nevertheless, the human auditory cortex is more complex than nonhuman primates (Hackett, 2015; Hackett et al., 2001) with multiple core and noncore areas of auditory cortex receiving thalamic inputs from different subdivisions of the medial geniculate complex (Jones, 2003; Burton and Jones, 1976; Winer and Schreiner, 2010), and early activity observed in HG could potentially reflect direct activation from auditory thalamus as opposed to that of intracortical synapses (Nourski et al., 2014). As such, a conclusive separation of core vs noncore areas in humans requires simultaneous anatomical and functional analysis of HG.

### Spatial organization of joint tuning properties

While the majority of previous research has examined the spatial organization of individual and isolated acoustic attributes, neurons in the mammalian auditory cortex have complex and multifeatured tuning properties (Bizley et al., 2013; King and Nelken, 2009; Walker et al., 2011). It is therefore crucial to examine the joint distribution of tuning properties to gain a more complete understanding of auditory field organization, particularly because a single tuning dimension may yield ambiguous separation of auditory fields (Barton et al., 2012). Here, we adopted an unbiased and unsupervised approach to find the primary and secondary correlational structure of tuning to various acoustic attributes. Our analysis uncovered two anatomical directions. The first direction runs from posterior-medial to anterior-lateral HG (labeled along HG in Fig. 2) and shows a gradient of change characterized by tuning to progressively lower frequencies and temporal modulation rates, increased latencies, speech sensitivity, and speaker invariance. The second axis, which was orthogonal to the previous HG axis (labeled across HG in Fig. 2), showed a gradient of change in frequency (low to high to low) and response latency. As such, we found both directions, medial to lateral and posterior to anterior, to be important in capturing the change in multidimensional acoustic feature tuning in HG. Although these characteristic maps share the same direction of change, additional analysis revealed the characteristic maps to be independent (Fig. S13).

Relating the functional properties of neural responses in HG to the underlying anatomy remains challenging. The direction of functional change that we observed along HG is consistent with the direction of anatomical gradients that are found using combined cyto- and receptor architectonic maps (Morosan et al., 2001, 2005). These structural studies divided HG into three areas, Te1.1, Te1.0 and Te1.2, which extend along the HG axis. The second direction of change, orthogonal to HG, is also consistent with anatomical changes, where the Te.1 region is surrounded by Te2.1 and TI on its sides. On the other hand, while a number of structural studies have shown left dominant asymmetry in the volume of HG (Morosan et al., 2001), we did not find a difference between the functional properties of left and right HGs in processing acoustic attributes. In summary, our results provide a comprehensive view of multidimensional acoustic processing in HG and pave the way towards a more complete functional characterization of auditory fields in the human auditory cortex.

## Supplementary Material

Supp material

## Figures and Tables

**Table 1:** Demographics, language laterality and seizure focus information. The age, sex, language laterality, seizure focus location, number of contacts and anatomical classification of HG are shown for each subject. No subject had a seizure focus in HG. The language-laterality was based on the WADA test. The number of contacts shows the number of contacts that were in the HG area and were responsive to speech. For anatomical classification, number 1 represents anatomical structure containing one single, smooth HG. Number 2 represents partially divided HG, meaning that it has lateral sulcus intermedius, but a common stem is intact. Number 3 represents fully divided HG. No subject showed a fully divided HG.

Subject	Age	Sex	Language laterality	Seizure focus Side	Location	Number of contacts		Anatomy type	
						Right	Left	Right HG	Left HG
1	46	M	L	Bilateral (left *>* right)	Superior temporal gyrus	7	8	2	2
2	59	M	L	Right	Mesial temporal	15	6	1	2
3	32	F	Not Tested	Bilateral (left *>* right)	Mesial temporal	8	11	2	2
4	31	F	L	Bilateral (left *>* right)	Mesial temporal	5	9	2	2
5	37	F	Bilateral (L *>* R)	Left	Basal temporal	5	0	1	1
6	32	F	L	Right	Mesial temporal and superior temporal gyrus	13	7	2	1
7	57	M	L	Right	Mesial temporal	8	4	1	1
8	33	F	R	Left	Frontal mesial	12	14	1	1
