# Supplementary material for: Functional characterization of human Heschl’s gyrus in response to natural speech
Source: Neuroimage. Author manuscript; Available in PMC 2021 Nov 22. (PMC8608271; doi:10.1016/j.neuroimage.2021.118003)
Supplement: Supp material [file NIHMS1731878-supplement-Supp_material.pdf]

Supplementary Information for

Functional characterization of human Heschl's gyrus in response to natural speech

Authors: Bahar Khalighinejad[1,2], Prachi Patel[1,2], Jose L. Herrero[3,4], Stephan Bickel[3,4], Ashesh D. Mehta[3,4], Nima Mesgarani[1,2*]

Affiliations:

[1]*Mortimer B. Zuckerman Mind Brain Behavior Institute, Columbia University, New York, NY, United States*

[2]*Department of Electrical Engineering, Columbia University, New York, NY, United States*

[3]*Hofstra Northwell School of Medicine, Manhasset, NY, United States*

[4]*The Feinstein Institutes for Medical Research, Manhasset, NY, United States*

* Correspondence to: nima@ee.columbia.edu

This PDF file includes:

Figs. S1 to S15

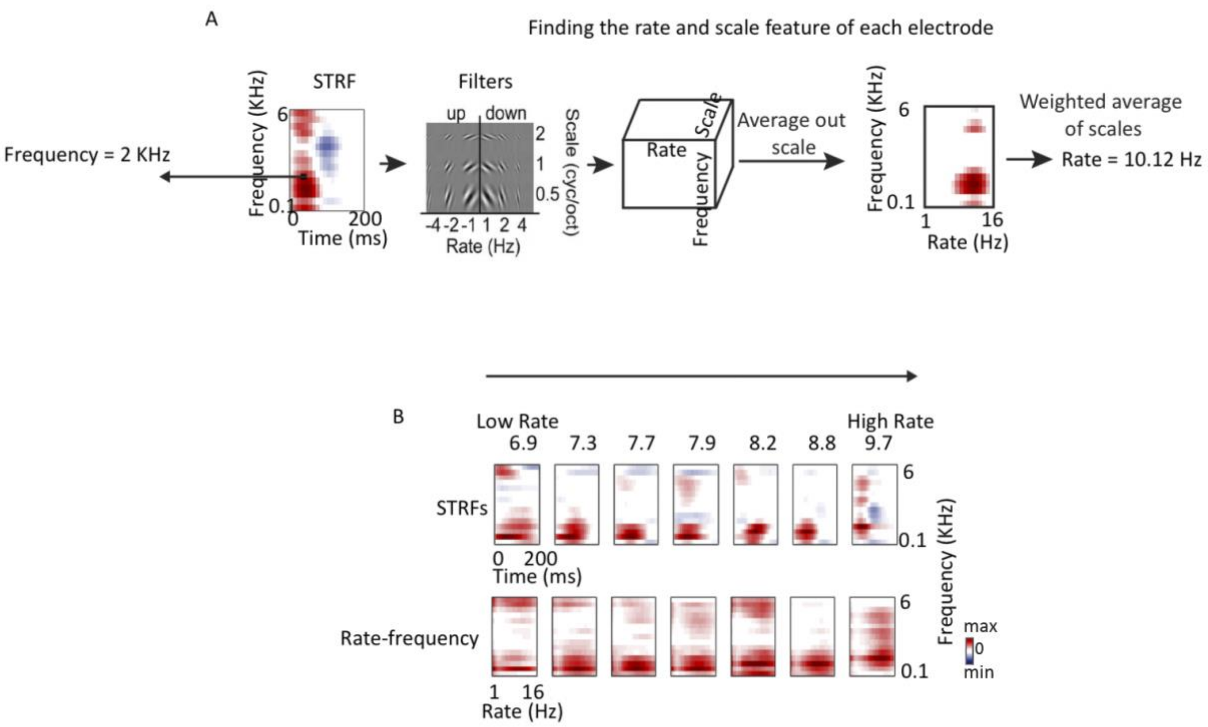

Supplementary figure 1. Calculating rate and frequency tuning properties from STRFs.

A) A computational model of the auditory cortex (Chi. Et. al. 2005) was used to find the temporal (rate) and spectral (scale) modulation representation of the STRFs. This transformation was performed by decomposing the STRFs using 2D wavelet transforms varying along the rate and scale dimensions.

B) Example STRFs sorted based on their best rate values. STRFs with higher and lower rates are more sensitive to fast and slow acoustic changes, respectively.

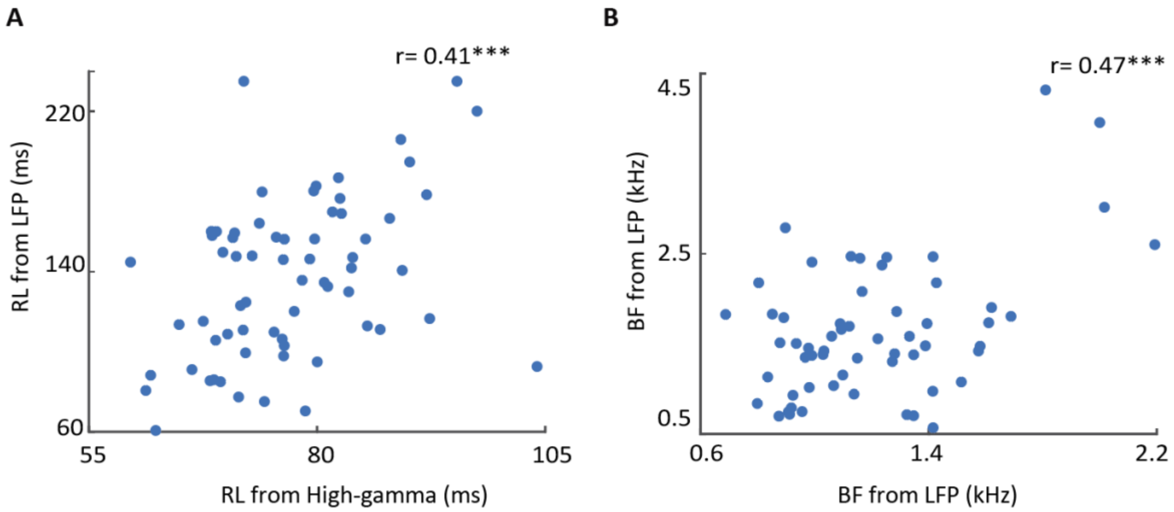

Supplementary figure 2. Relationship between high gamma activity and LFP.

(A) Scatter of response latency from LFP and high gamma activity for single electrodes.

(B) Scatter of best frequency from LFP and high gamma activity for single electrodes.

A

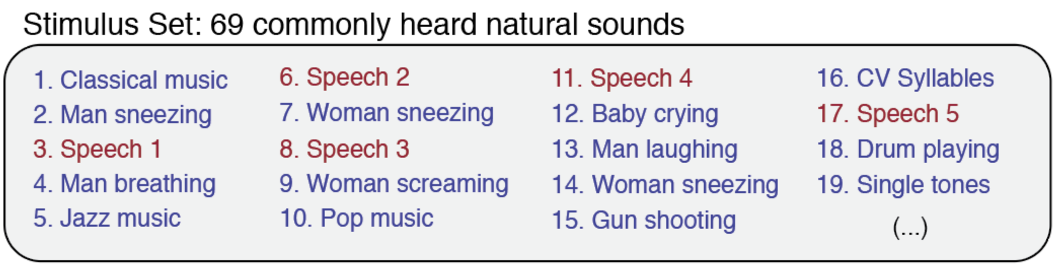

Supplementary figure 3. Speech-specificity stimulus set.

53 sounds were non-speech, 16 were speech.

The complete list of categories of nonspeech sounds is as follows:

1. Coughing
2. Crying
3. Screaming
4. Music (Jazz, Pop, Classical)
5. Animal vocalization
6. Laughing
7. Syllables
8. Sneezing
9. Breathing
10. Singing
11. Shooting
12. Tones
13. Drum playing
14. Subway noise

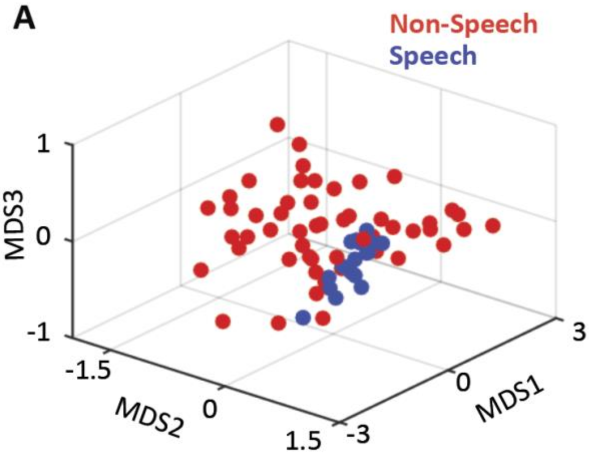

Supplementary figure 4. MDS for speech and non-speech stimuli based on the differences in spectrograms.

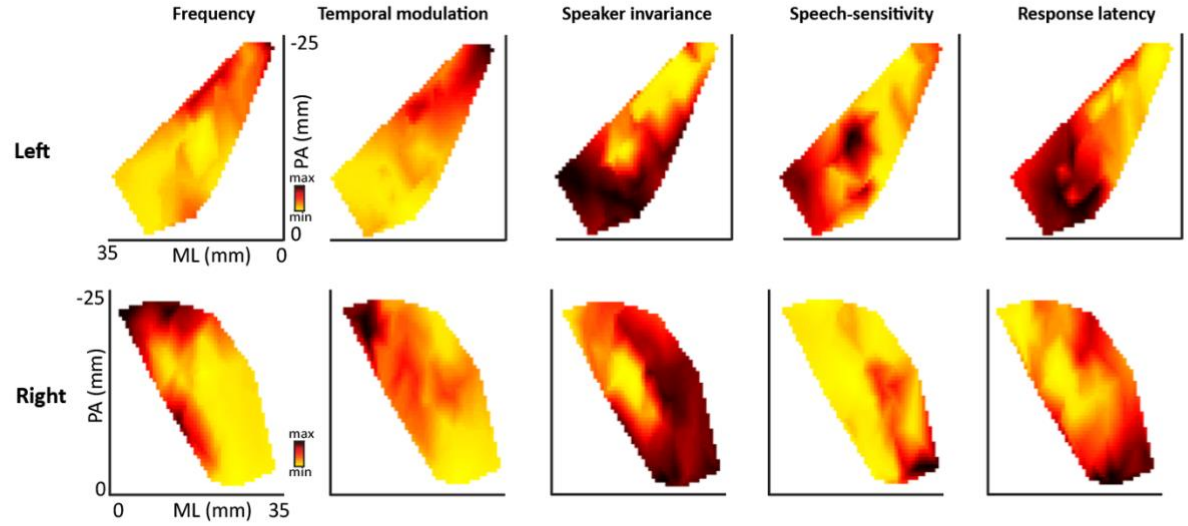

Supplementary figure 5. Characterization maps of left and right Heschl's gyrus.

The five characteristic maps of best frequency, best temporal modulation, speaker invariance index, speech sensitivity and response latency are shown for the left hemisphere (top row) and right hemisphere (bottom row).

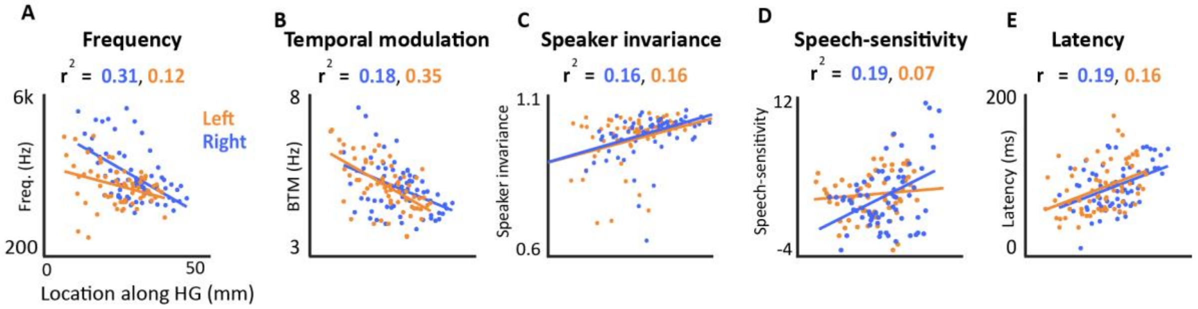

Supplementary figure 6. Along Heschl's gyrus (HG) gradient for left vs right Heschl's gyrus.

A to E) Frequency, temporal modulation, speaker invariance, speech sensitivity, and response latency for different neural sites are shown on the Y-axis, and the location along HG is shown on the X-axis. Orange indicates the neural sites on the left HG, and blue indicates the neural sites on the right HG.

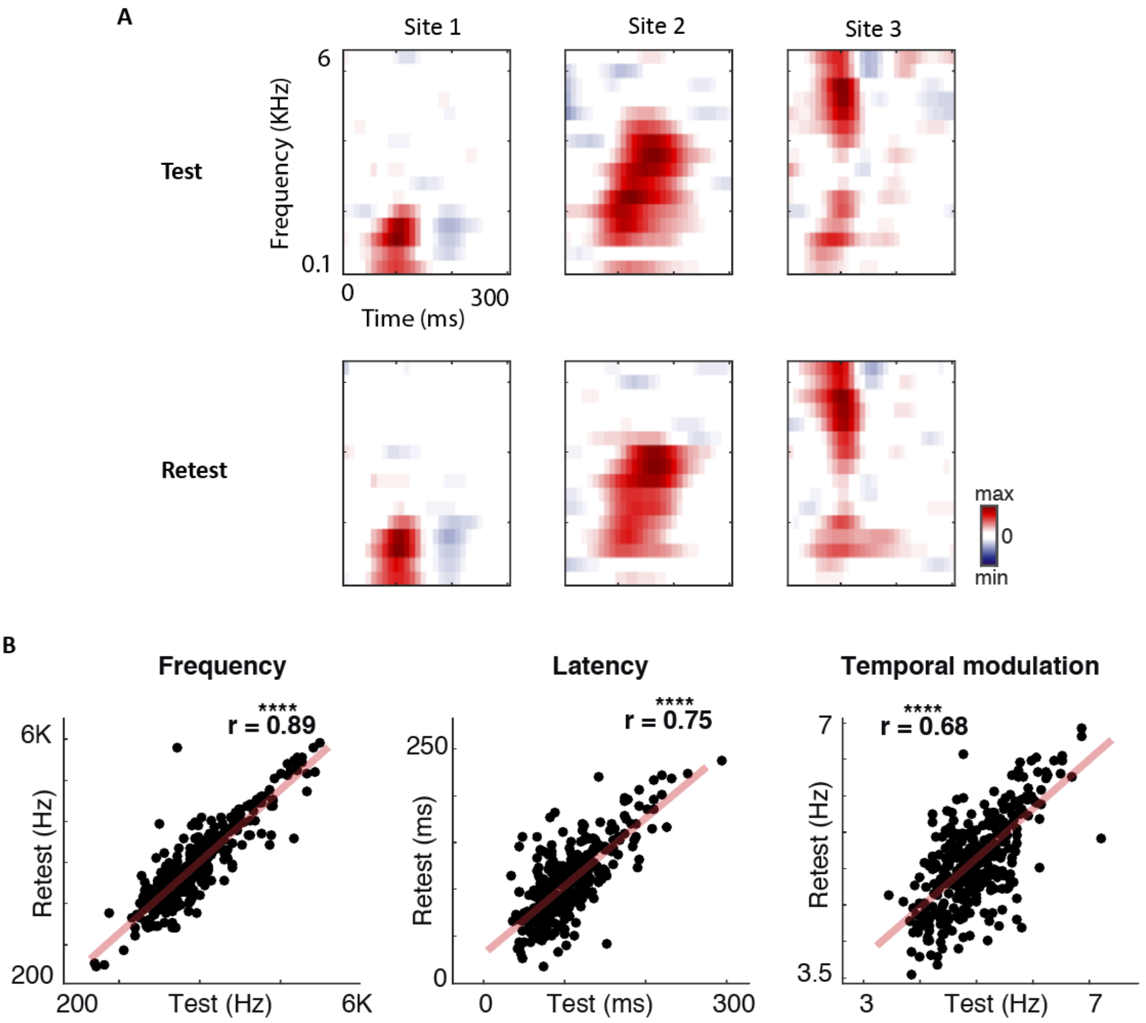

Supplementary figure 7. Test-retest reliability of STRFs.

Finding STRFs based on a test-retest reliability test, the stimuli and responses were segmented into two nonoverlapping subsets and STRFs were calculated from each subset separately. A few example STRFs from test and retest are shown in (A) where the top row shows the test and the bottom row shows the retest. (B) Correlation between test and retest values for best frequency, latency and temporal modulation.

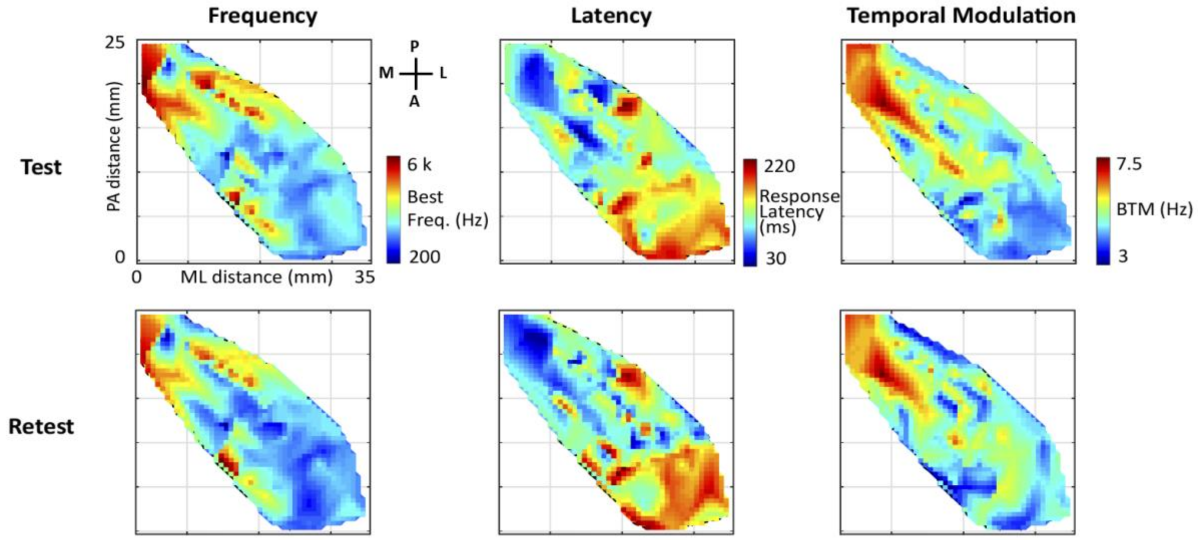

Supplementary figure 8. Characteristic maps using test and retest stimuli.

Characteristic maps calculated based on the test (top row) and retest (bottom row) are highly similar.

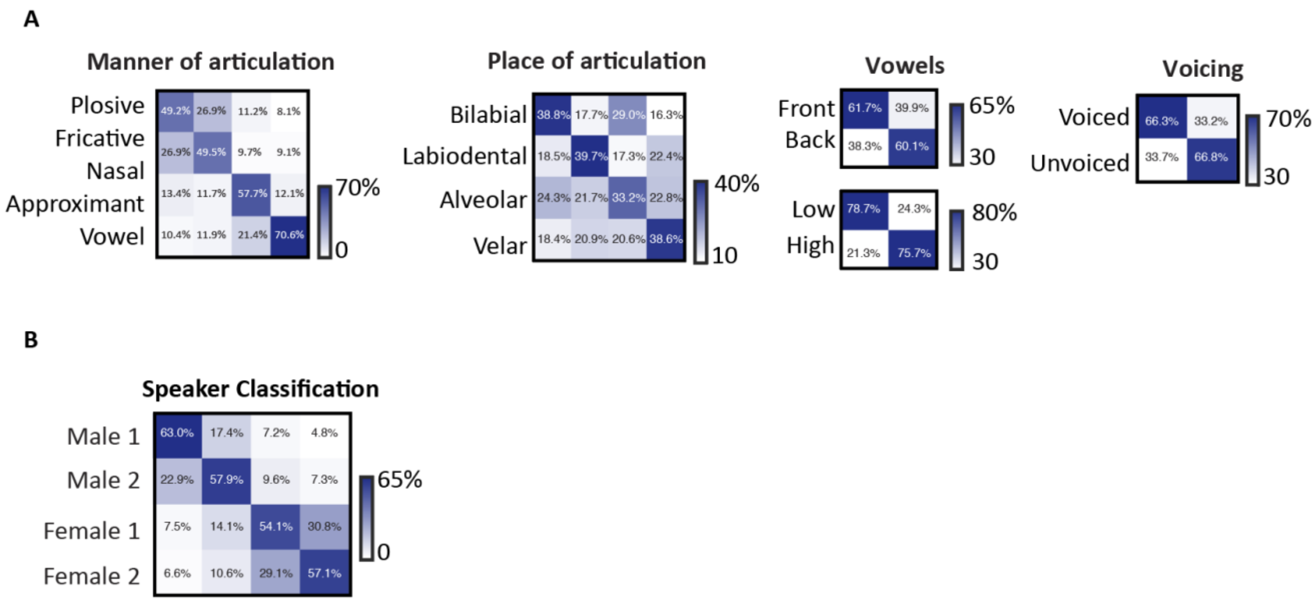

Supplementary figure 9. Confusion matrices for classification analysis.

(A) Confusion matrices for phonetic decoding.

(B) Confusion matrices for speaker identity decoding.

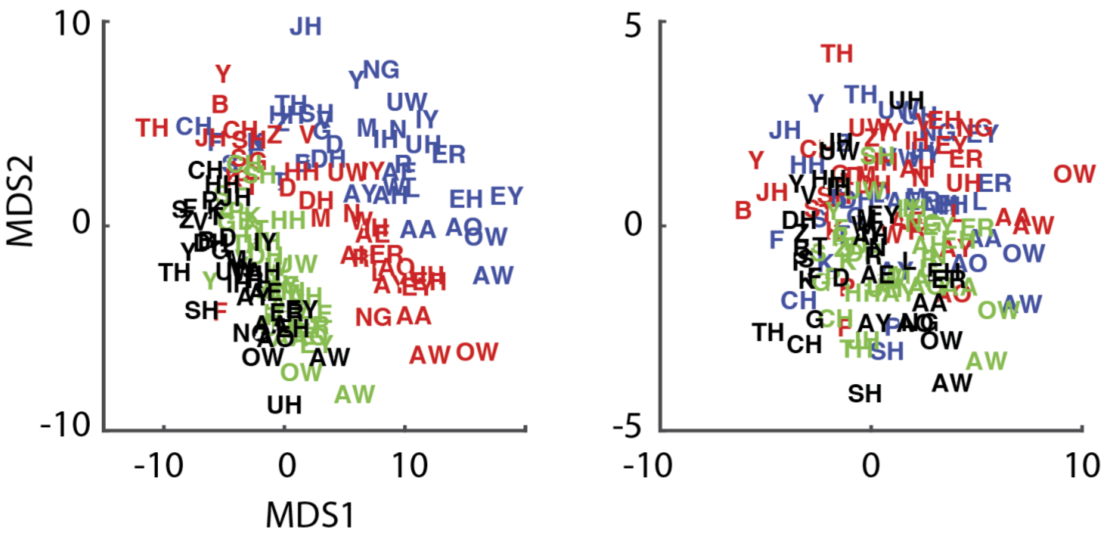

Supplementary figure 10. MDS diagram of all phonemes for neural responses in the PM and AL areas.

The posteromedial (PM) area is shown on the left, and the anterolateral (AL) area is shown on the right. The four colors indicate four different speakers where black is male 1, green is male 2, red is female 1, and blue is female 2.

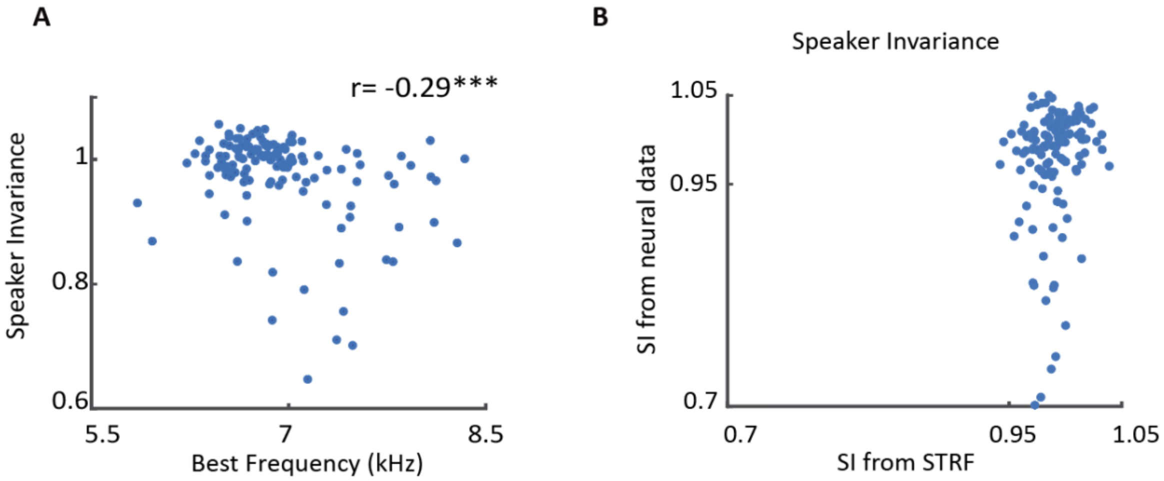

Supplementary figure 11.

(A) Scatter showing the relationship between the best frequency map and speaker invariance map.

(B) The scatter of speaker invariance from actual neural data and from the neural data predicted using STRF shows a larger range of SI from neural compared to STRF predicted data.

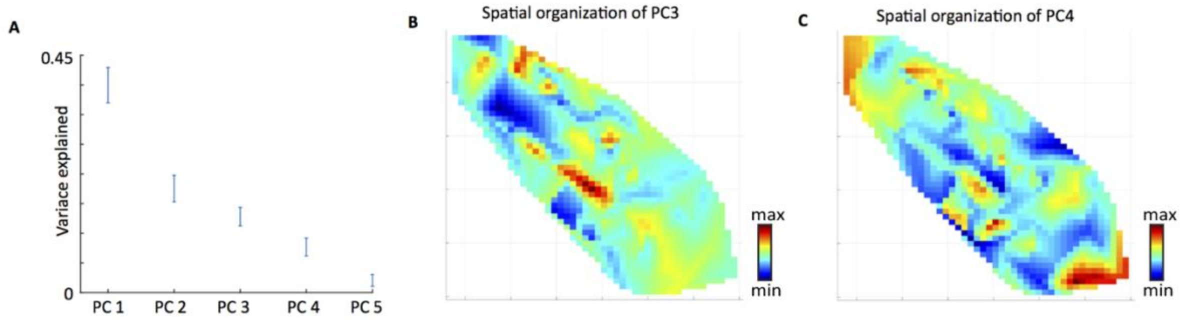

Supplementary figure 12. Principal component analysis of joint functional characteristics.

(A) Variance explained by PCs 1 to 5 is shown.

(B, C) There was no simple spatial arrangement for PC 3 and PC 4.

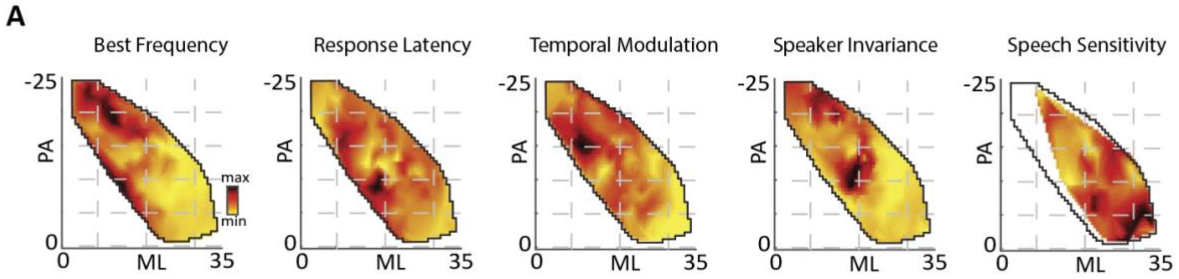

Supplementary figure 13. Standard deviation map for each of the characteristic feature maps. The standard deviation is measured across every four neighboring electrodes in the aggregated map of all subjects. Each characteristic map has a different location of high standard deviation showing absence of a unified direction of change.

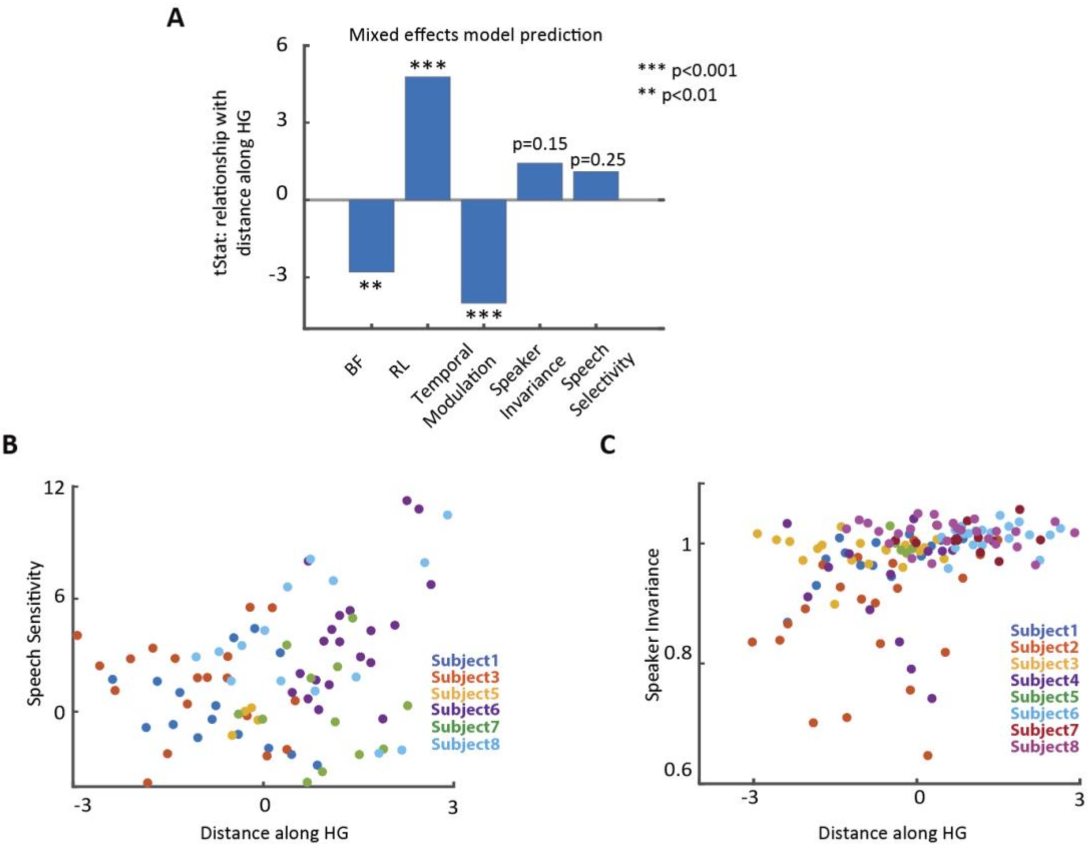

Supplementary figure 14. Results controlling for individual subject identity.

(A) Mixed effect linear model prediction controlling for subject identity as a random effect of a mixed effect linear model. The bar plot shows the relationship of the 5 features from the paper and the distance along HG.

(B) Scatter of speech sensitivity with distance along HG (left) and speaker invariance and distance along HG. Each dot is a single electrode colored by subject number.

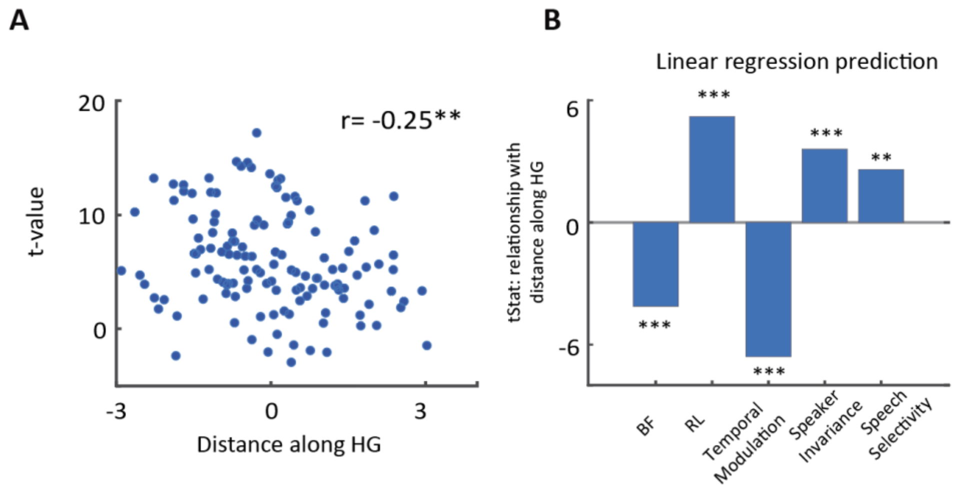

Supplementary figure 15. Results controlling for high gamma activity responsiveness effects.
(A) Scatter of t-values indicating high gamma responsiveness and distance along HG for single electrodes.
(B) Linear regression analysis controlling for the t-value and verifying the relationship between features and distance along HG.
